# Diminished activity-dependent BDNF signaling differentially causes autism-like behavioral deficits in male and female mice

**DOI:** 10.3389/fpsyt.2023.1182472

**Published:** 2023-05-03

**Authors:** Kaijie Ma, Connie Taylor, Mark Williamson, Samuel S. Newton, Luye Qin

**Affiliations:** ^1^Division of Basic Biomedical Sciences, Sanford School of Medicine, University of South Dakota, Vermillion, SD, United States; ^2^Department of Pediatrics, Sanford School of Medicine, University of South Dakota, Sioux Falls, SD, United States; ^3^Biostatistics, Epidemiology, and Research Design Core, University of North Dakota, Grand Forks, ND, United States

**Keywords:** autism spectrum disorder, activity-dependent neural signaling, BDNF, Val66Met, anxiety, social behaviors, sex, cognition

## Abstract

Autism spectrum disorder (ASD) is a group of neurodevelopmental disorders with strong genetic heterogeneity and more prevalent in males than females. Recent human genetic studies have identified multiple high-risk genes for ASD, which produce similar phenotypes, indicating that diverse genetic factors converge to common molecular pathways. We and others have hypothesized that activity-dependent neural signaling is a convergent molecular pathway dysregulated in ASD. However, the causal link between diminished activity-dependent neural signaling and ASD remains unclear. Brain-derived neurotrophic factor (BDNF) is a key molecule mediating activity-dependent neural signaling. We therefore hypothesize that diminished activity-dependent BDNF signaling could confer autism-like behavioral deficits. Here, we investigated the effect of diminished activity-dependent BDNF signaling on autism-like behavioral deficits by using mice with genetic knock-in of a human BDNF methionine (Met) allele, which has decreased activity-dependent BDNF release without altering basal BDNF level. Compared with wild-type (WT) controls, diminished activity-dependent BDNF signaling similarly induced anxiety-like behaviors in male and female mice. Notably, diminished activity-dependent BDNF signaling differentially resulted in autism-like social deficits and increased self-grooming in male and female mice, and male mice were more severe than female mice. Again, sexually dimorphic spatial memory deficits were observed in female BDNF^+/Met^ mice, but not in male BDNF^+/Met^ mice. Our study not only reveals a causal link between diminished activity-dependent BDNF signaling and ASD-like behavioral deficits, but also identifies previously underappreciated sex-specific effect of diminished activity-dependent BDNF signaling in ASD. These mice with genetic knock-in of the human BDNF Met variant provide a distinct mouse model for studying the cellular and molecular mechanisms underlying diminished activity-dependent neural signaling, the common molecular pathway dysregulated in ASD.

## 1. Introduction

Autism spectrum disorder (ASD) is a group of neurodevelopmental disorders characterized by persistent deficits in social communication and interaction, and restricted and repetitive patterns of behavior, interests, or activities [American Psychiatric Association (2013): Diagnostic and Statistical Manual of Mental Disorders, 5th edition]. ASD affects 1 in 44 children with a ratio of about 1 female for every 4 males in the US (CDC: Centers for Diseases Control and Prevention). A limitation of research in ASD has been its importance on males, which precludes our understanding of autism in females and highlights the need to study the neural mechanisms of ASD in both sexes.

Recent large-scale human genetic studies have identified many risk genes for ASD, which confirm that ASD has a strong genetic heterogeneity (Simons Foundation Autism Research Initiative, SFARI) (1, 2). Those risk genes induce similar autism phenotypes, which indicates common molecular pathways likely exist. Growing evidence suggests that activity-dependent neural signaling is a common molecular pathway dysregulated in ASD caused by multiple genetic mutations (3, 4), such as *GRIN2B* and *SHANK3*. NR2B (encoded by *GRIN2B*) is one of the major subunits of N-methyl-D-aspartate (NMDA) receptors. NMDA receptors mediated signaling is required for neuronal survival, synaptic plasticity, social behaviors (5) and memory formation (6). NMDA receptor subunit variants were implicated in ASD (2, 7–10). Administration of NMDA receptor antagonists (11, 12) or NR1 (one of the subunits of NMDA receptors) deficiency induced ASD-like social deficits in mice (13–15). SHANK3 is a scaffolding protein at postsynaptic density (PSD) of glutamatergic synapses. Haploinsufficiency of the *SHANK3* due to deletions or *de novo* mutations has been linked to autism (2, 16, 17). Ours and others’ studies have demonstrated that *Shank3*-deficiency significantly diminished NMDA receptors-and AMPA (α-amino-3-hydroxy-5-methyl-4-isoxazolepropionic acid) receptors-mediated glutamatergic synaptic transmission (5, 12, 18), which secondarily diminish activity-dependent neural signaling (5). However, the causality of diminished activity-dependent neural signaling in ASD remains largely unknown.

Activity-dependent neural signaling is crucial for synaptic development, plasticity, and function (3, 4), which has drawn our attention to the central role played by brain-derived neurotrophic factor (BDNF). BDNF is a key mediator of activity-dependent processes *via* binding to two membrane receptors: tyrosine kinase receptor B (TrkB) and low affinity neurotrophic factor receptor (also known as p75). Homozygous offspring of whole-body deletion of *Bdnf* lead to lethality, suggesting its essential role in embryonic brain development (19, 20). However, BDNF has particular roles in glutamatergic and GABAergic transmission, synaptic connections, synapse structure, neurotransmitter release, and synaptic plasticity during postnatal neuronal development (21, 22). A common single nucleotide polymorphism (SNP) in the pro-domain of human *BDNF* gene that leads to a methionine (Met) substitution for valine (Val) at codon 66 (Val66Met) significantly reduces dendritic trafficking, synaptic localization of the protein, and decreases up to 30% of activity-dependent BDNF release without affecting basal BDNF secretion (23, 24). The behavioral impact of diminished activity-dependent BDNF signaling on autism has not been evaluated and should be addressed to demonstrate its etiological role in ASD.

In this study, we characterized the impact of diminished activity-dependent BDNF signaling on autism-like behavioral deficits by using male and female mice with knock-in of a human BDNF Met allele to model individuals with decreased activity-dependent neural signaling caused by diverse genetic abnormalities in patients with ASD.

## 2. Materials and methods

### 2.1. Animal care and husbandry

The use of animals and procedures performed were approved by the Institutional Animal Care and Use Committee of Sanford School of Medicine, University of South Dakota. A mouse model with genetic knock-in of a human BDNF Met variant was created and the procedures for heterozygote breeding and genotyping were described previously (23). These mice were backcrossed more than 12 generations into the C57BL/6 strain. Animals were group-housed (*n* = 4–5) in standard cages and were kept on a 12-h light–dark cycle in a temperature-controlled room. Food and water were available *ad libitum*. Experiments were performed in male and female heterozygous BDNF^+/Met^ mice and sex-and age-matched WT littermates BDNF*^+/+^*, which were derived from heterozygous BDNF^+/Met^ breeding pairs. All behavioral assays were performed when mice were 6–7 weeks old. Five cohorts of male and female mice were used to reach the final sample sizes. Experiments were carried out by investigators in a blinded fashion (with no prior knowledge of genotypes). The behavioral tests were performed according to the order below.

### 2.2. Open field test

Animals were placed in an apparatus (L: 67.7 cm; W: 50.8 cm; H: 50.8 cm) to move freely for 10 min. The total distance traveled and the amount of time the animal spent in the center (29 cm × 20 cm) were counted by Ethovision XT tracking software (Noldus, Leesburg, VA).

### 2.3. Elevated plus maze (EPM) test

The EPM test was used to assess anxiety-like behaviors under stress (25). Mice were placed in the center of a plus maze that was elevated 50 cm above the floor with two opposite open arms and two opposite closed arms (each arm was 88 cm long, and 28 cm-height walls only on the closed arms) arranged at right angles. The number of entries and time spent in the closed and open arms were monitored for 10 min. The behaviors were video recorded and automatically scored using Ethovision XT tracking software (Noldus, Leesburg, VA).

### 2.4. Social preference test

A three-chamber social interaction assay was performed to assess social deficits (5). Briefly, an apparatus (L: 101.6 cm; W: 50.8 cm; H: 50.8 cm) containing three chambers with retractable doorways allowing for access to side chambers was used. Animals were habituated to the apparatus for 1 day before testing. During the habituation, two empty capsules (inverted pencil cup, D: 10.2 cm, H: 10.5 cm) were placed in the corner of the chambers, and an upright cup was placed on top of each capsule to prevent the subject mouse from climbing on top. Animals were allowed to explore all three chambers of the apparatus for 10 min. The test was composed of two phases with different stimuli in each of the side chambers. The phase 1 contained two identical nonsocial stimuli (folded papers), and the phase 2 contained a nonsocial (NS) stimulus (a woodblock) and a social (Soc) stimulus (an age-, sex-, strain-matched mouse). Each stimulus was placed inside a capsule placed in the corner of the chamber. The test animal was placed in the center chamber and was free to explore the apparatus for 10 min in each phase, while it was returned to its home cage during the 10-min intervals between phases. The chamber was cleaned with 75% ethanol after each phase. Interaction time was counted based on the “investigating” behaviors of the test animal to each stimulus. A computer running Ethovision XT tracking software (Noldus, Leesburg, VA) measured the time of the test animal spent at the proximity of the capsule (distance of animal head to cup edge: ≤3.5 cm). Preference index scores were calculated, where time spent with one stimulus was subtracted from the time spent with the other stimulus and divided by the total time spent exploring both stimuli.

### 2.5. Self-grooming

Mice were scored for spontaneous grooming behaviors when placed individually in a clean cage. The cage was lined with a thin layer of bedding (~1 cm) to reduce neophobia but prevent digging, a potentially competing behavior. Prior to the testing period, animals were allowed to habituate to the novel environment for 10 min. Each mouse was rated for 10 min on cumulative time spent grooming (5).

### 2.6. Barnes maze test

Barnes maze test was used to measure spatial memory (26, 27). Mice were placed on a round platform with eight equally spaced holes at the edge, one of which was attached with an escape box (correct hole). Bright overhead light was applied as a weak aversive stimulation to increase the motivation to escape from the circular platform. During the two learning phases (5-min interval) (information acquisition), mice were allowed to explore the platform using distal visual cues until finding the correct hole and entering the escape box. Then, mice were placed in its home cage to rest for 15 min. In the memory phase (information retention and retrieval), the escape box was removed, and mice were put back on the platform to explore for 5 min. Mice spent the time with their noses oriented toward the hole within 3.5 cm of the hole edge was considered. The time spent on the correct hole (T1) and the other seven incorrect holes (T2) were counted. Spatial memory index was calculated by T1/T2. The behaviors were video recorded and automatically scored using Ethovision XT tracking software (Noldus, Leesburg, VA).

### 2.7. Novel object recognition test

The NOR test was used to assess object recognition memory (27, 28). The test was composed of three phases: habituation (no objects), familiarization (two identical objects “familiar-A,” 5 min), and test [(familiar-A) and a new, different object (“novel-B”), 5 min] separated by a short delay period (5 min). The room was illuminated by indirect white light. All objects were made of plastic toys (height, about 5 cm) with similar textures, colors, and sizes but distinctive shapes. The mouse was removed from the arena and placed in its holding cage at each interval between phases. During habituation, mice were placed into the open field apparatus (L: 67.7 cm, W: 50.8 cm, H: 50.8 cm) for 5 min. During familiarization, two identical objects were placed in the opposite corners counterbalanced, and the animals were allowed to explore the objects for 5 min. During test phase, mice were placed in the same apparatus, one object of the pair was replaced with a novel object, and they were allowed to freely explore for 5 min. Mice spent the time with their nose oriented toward the object within 3.5 cm of the object’s edge, and/or touching it with the nose was considered, but not sitting on the object. Total exploration time of the familiar and novel objects was recorded. The discrimination index was calculated as: [time spent on novel object (B) − time spent on familiar object (A)]/[total time exploring both objects (B + A)] for the test session. The behaviors were video recorded and automatically scored using Ethovision XT tracking software (Noldus, Leesburg, VA).

### 2.8. Rotarod test

To assess motor coordination and balance, an accelerating rotarod (San Diego Instruments, San Diego CA) was used. Mice were placed on a cylinder, which slowly accelerated from 4 to 40 r.p.m. over a 5-min test session. The task requires mice to walk forward to remain on top of the rotating cylinder rod.

### 2.9. Statistical analysis

To detect behavioral differences in mice, sample size was calculated based on predicting detectable differences to reach power of 0.80 at a significance level of 0.05 by running power analyses in G*Power software. For two-way ANOVA (numerator df = 1, number of groups = 4), based on our previous studies, we designed effect sizes of 2.48 down to 1.08. Therefore, we will use 10 mice per group. Additional descriptive statistics and statistical comparisons will be performed using GraphPad software Prism 7.0 (GraphPad Software, La Jolla, CA). For statistical significance, experiments with 4 groups were subjected to a two-way ANOVA followed by *post hoc* Bonferroni tests for multiple comparisons. Data were presented as mean ± SEM.

## 3. Results

### 3.1. Diminished activity-dependent BDNF signaling similarly induces anxiety-like behaviors in male and female mice

**Figure 1:**
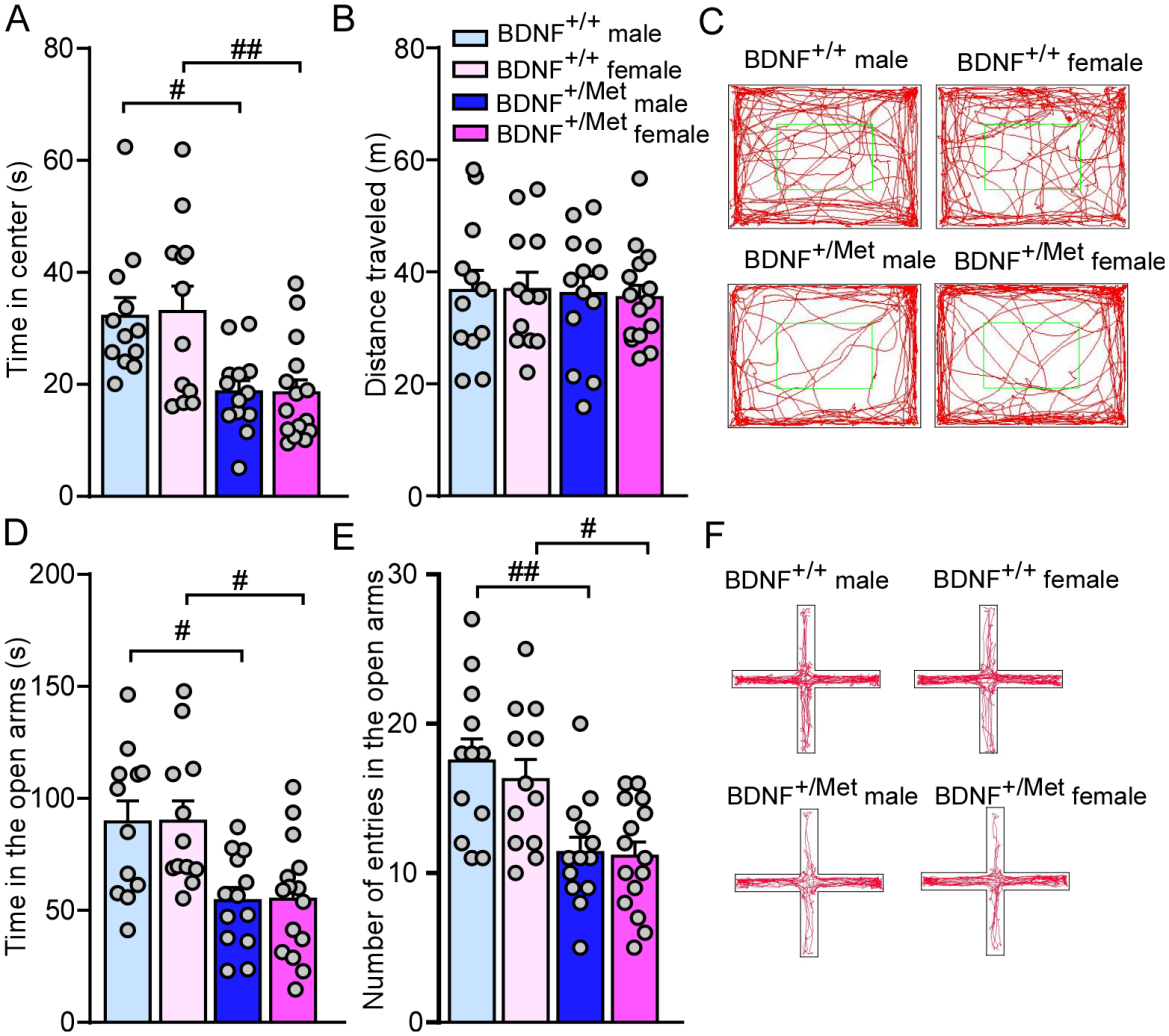
Diminished activity-dependent BDNF signaling similarly induces anxiety-like behaviors in males and females. **(A,B)** Bar graphs showing time spent in center and total distance traveled during open field test of male and female BDNF^+/+^ and BDNF^+/Met^ mice. **(C)** Representative trajectory diagrams of mice in the open field test. **(D,E)** Bar graphs showing time spent and number of entries in the open arms during EPM test of male and female BDNF^+/+^ and BDNF^+/Met^ mice. **(F)** Representative trajectory diagrams of mice in the EPM test. ^#^*p* < 0.05, ^##^*p* < 0.01, BDNF^+/Met^ vs. BDNF^+/+^. Male BDNF^+/+^ mice: *n* = 12; Female BDNF^+/+^ mice: *n* = 12; Male BDNF^+/Met^ mice: *n* = 13; Female BDNF^+/Met^ mice: *n* = 15.

The human BDNF Met allele knock-in mouse model was created and described in our previous publications (23, 29, 30). We have used heterozygous BDNF^+/Met^ mice, which models ASD patients with haploinsufficiency of diverse genetic factors. Anxiety is a common comorbidity of ASD, which affects social ability. It has been reported that male and female homozygous BDNF^Met/Met^ mice displayed anxiety-like behaviors in the open field test (23, 31). It is unknown how sex interacts with heterozygous mice with diminished activity-dependent BDNF signaling in anxiety. As shown in Figures 1A–C, male and female BDNF^+/Met^ mice displayed less time (*F*_Genotype (1, 48)_ = 20.3, *p* < 0.0001; *F*_Sex (1, 48)_ = 0.01, *p* = 0.92, Figure 1A) spent at the center in the open field test. The comparable distance traveled (*F*_Genotype (1, 48)_ = 0.1, *p* = 0.75; *F*_Sex (1, 48)_ = 0.009, *p* = 0.93, Figure 1B) in the open field test and similar latency to fall (*F*_Genotype (1, 48)_ = 0.04, *p* = 0.83; *F*_Sex (1, 48)_ = 0.01, *p* = 0.92, Supplementary Figure 1) during rotarod test demonstrated that diminished activity-dependent BDNF signaling induced anxiety-like behaviors in both males and females without affecting locomotion activity and movement coordination. To further examine diminished activity-dependent BDNF signaling similarly induces anxiety-like behaviors in males and females, we performed an elevated plus maze (EPM) test to examine anxiety-like behaviors under a stress condition (25). Compared to BDNF^+/+^ controls, male and female BDNF^+/Met^ mice spent relatively less time (*F*_Genotype (1, 48)_ = 20.43, *p* < 0.0001, *F*_Sex (1, 48)_ = 0.005, *p* = 0.94, Figure 1D) and number of entries (*F*_Genotype (1, 48)_ = 21.93, *p* < 0.0001, *F*_Sex (1, 48)_ = 0.39, *p* = 0.53, Figures 1E,F) in the open arms, confirming diminished activity-dependent BDNF signaling induced anxiety-like behaviors in both sexes under a stress condition. Therefore, these results suggest that diminished activity-dependent BDNF signaling similarly induces anxiety-like behaviors in males and females under relatively mild and high stress conditions.

### 3.2. Diminished activity-dependent BDNF signaling differentially causes autism-like behavioral deficits in male and female mice

**Figure 2:**
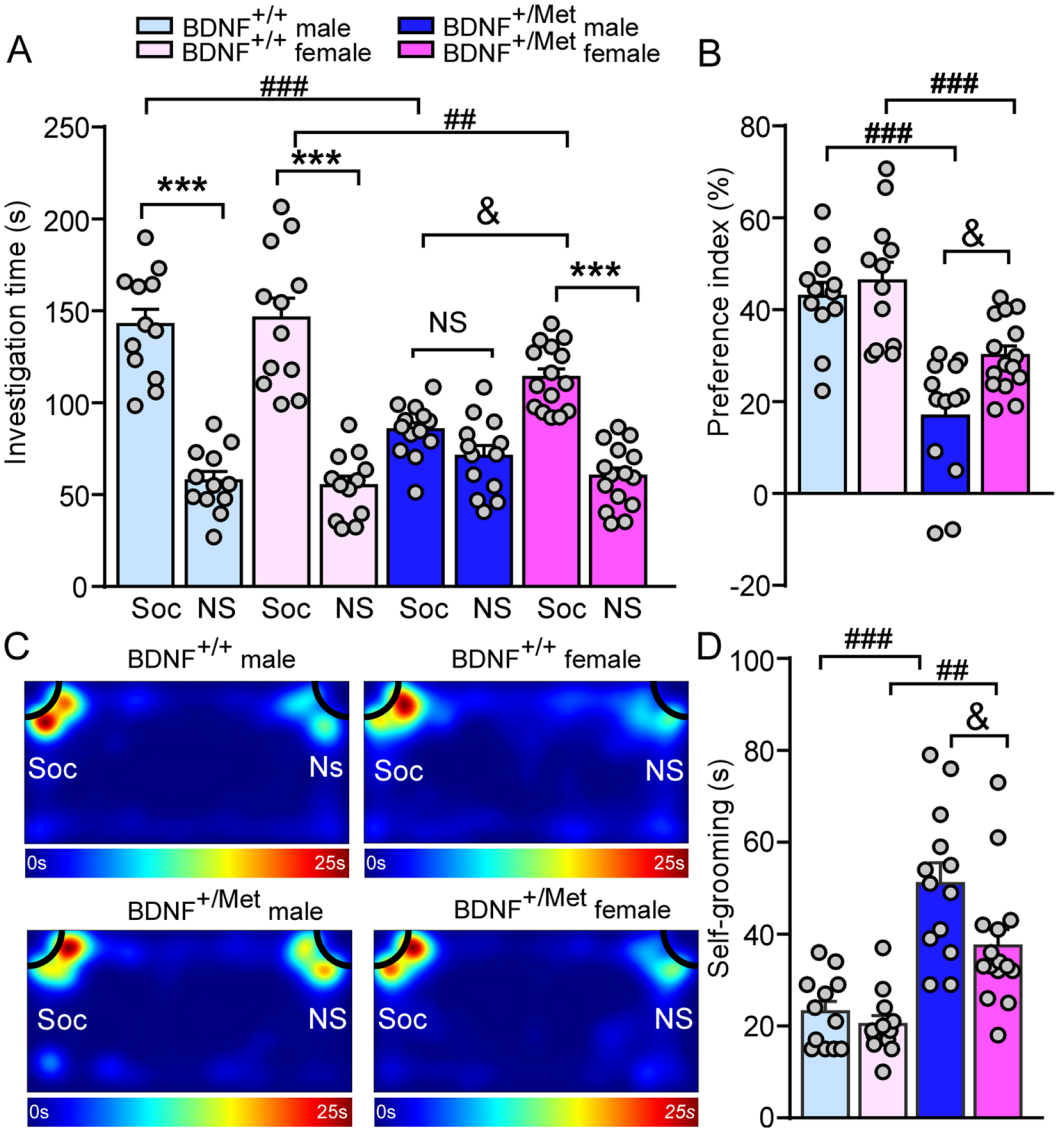
Diminished activity-dependent BDNF signaling differentially causes autism-like behavioral deficits in males and females. **(A,B)** Bar graphs showing the time spent investigating either the social (Soc) or non-social (NS) stimulus and social preference index in three-chamber sociability test of male and female BDNF^+/+^ and BDNF^+/Met^ mice. **(C)** Representative heatmaps illustrating the time spent in different locations of the three chambers from the social preference test. **(D)** Bar graphs showing the time spent self-grooming in male and female BDNF^+/+^ and BDNF^+/Met^ mice. ***p < 0.001, Soc vs. NS; ^##^*p* < 0.01, ^###^*p* < 0.001, BDNF^+/Met^ vs. BDNF^+/+^; ^&^*p* < 0.05, male vs. female. Male BDNF^+/+^ mice: *n* = 12; Female BDNF^+/+^ mice: *n* = 12; Male BDNF^+/Met^ mice: *n* = 13; Female BDNF^+/Met^ mice: *n* = 15.

To determine whether diminished activity-dependent BDNF signaling causes the core autism-like social behavioral deficits, male and female BDNF^+/Met^ mice, and sex-, age-matched BDNF^+/+^ mice were subjected to the three-chamber social interaction assay (5). Given the presence of anxiety-like deficits in BDNF^+/Met^ mice, social-and non-social stimuli were placed in the corners of two side chambers to minimize the interference of anxiety to social ability (32). The social interaction assay is composed of habituation and two phases with various stimuli placed in each of the two side chambers as before (5, 33). During phase I of two identical non-social stimuli (NS1-NS1), male and female BDNF^+/Met^ and BDNF^+/+^ mice spent similar time exploring the two identical non-social objects (*F*_Interaction (3, 96)_ = 0.54, *p* = 0.66, Supplementary Figure 2), which confirmed that there was no pre-existing side preference in both strains. In phase 2, male and female BDNF^+/+^ mice spent significantly more time exploring the social stimulus over the non-social object, while male BDNF^+/Met^ displayed no preference and female BDNF^+/Met^ displayed reduced preference for the social stimulus [Male BDNF^+/+^: social: 142.6 ± 8.4 s, nonsocial: 57.6 ± 5 s, *n* = 12; Female BDNF^+/+^: social: 146.1 ± 10.9 s, nonsocial: 55 ± 5.1 s, *n* = 12; Male BDNF^+/Met^ social: 85.3 ± 4.0 s, nonsocial: 71 ± 5.7 s, *n* = 13; Female BDNF ^+/Met^, social: 113.9 ± 4.6 s, nonsocial: 60 ± 4.4 s, *n* = 15, *F*_interaction (3, 96)_ = 15.75, *p* < 0.0001, two-way ANOVA, Figure 2A]. Consistently, male and female BDNF^+/Met^ displayed significantly reduced social preference index, compared with male and female BDNF^+/+^ mice [Male BDNF^+/+^: 43% ± 3%, *n* = 12; Female BDNF^+/+^: 46.3% ± 4.1%, *n* = 12; Male BDNF^+/Met^: 16.9% ± 3.7%, *n* = 13; Female BDNF^+/Met^: 30.1% ± 2%, *n* = 15. *F*_Genotype (1, 48)_ = 43.7, *p* < 0.0001, *F*_Sex (1, 48)_ = 6.6, *p* = 0.013, two-way ANOVA, Figures 2B,C]. There was a significantly lower social preference index in male than in female BDNF^+/Met^ mice, which suggests diminished activity-dependent BDNF signaling sexually differentially affects the severity of autism-like social deficits.

Repetitive and restrictive interests or activities are the second core symptoms of ASD. Self-grooming is an innate behavior in rodents and is used as an indication of compulsive and repetitive behavior (34). As shown in Figure 2D, male and female BDNF^+/Met^ mice spent significantly more time engaged in self-grooming, compared to male and female BDNF^+/+^ mice [Male BDNF^+/+^: 23.1 ± 2.3 s, *n* = 12; Female BDNF^+/+^: 20.3 ± 2 s, *n* = 12; male BDNF^+/Met^: 51 ± 4.5 s, *n* = 13, Female BDNF^+/Met^: 37.5 ± 3.6 s, *n* = 15. *F*_Genotype (1, 48)_ = 44.58, *p* < 0.0001, *F*_Sex (1, 48)_ = 5.82, *p* = 0.019, two-way ANOVA]. The significant higher time engaged in self-grooming in male BDNF^+/Met^ mice than female BDNF^+/Met^ mice indicates the autism-like repetitive and restrictive behaviors in males are more severe than those of in females.

### 3.3. Diminished activity-dependent BDNF signaling does not affect object recognition memory

**Figure 3:**
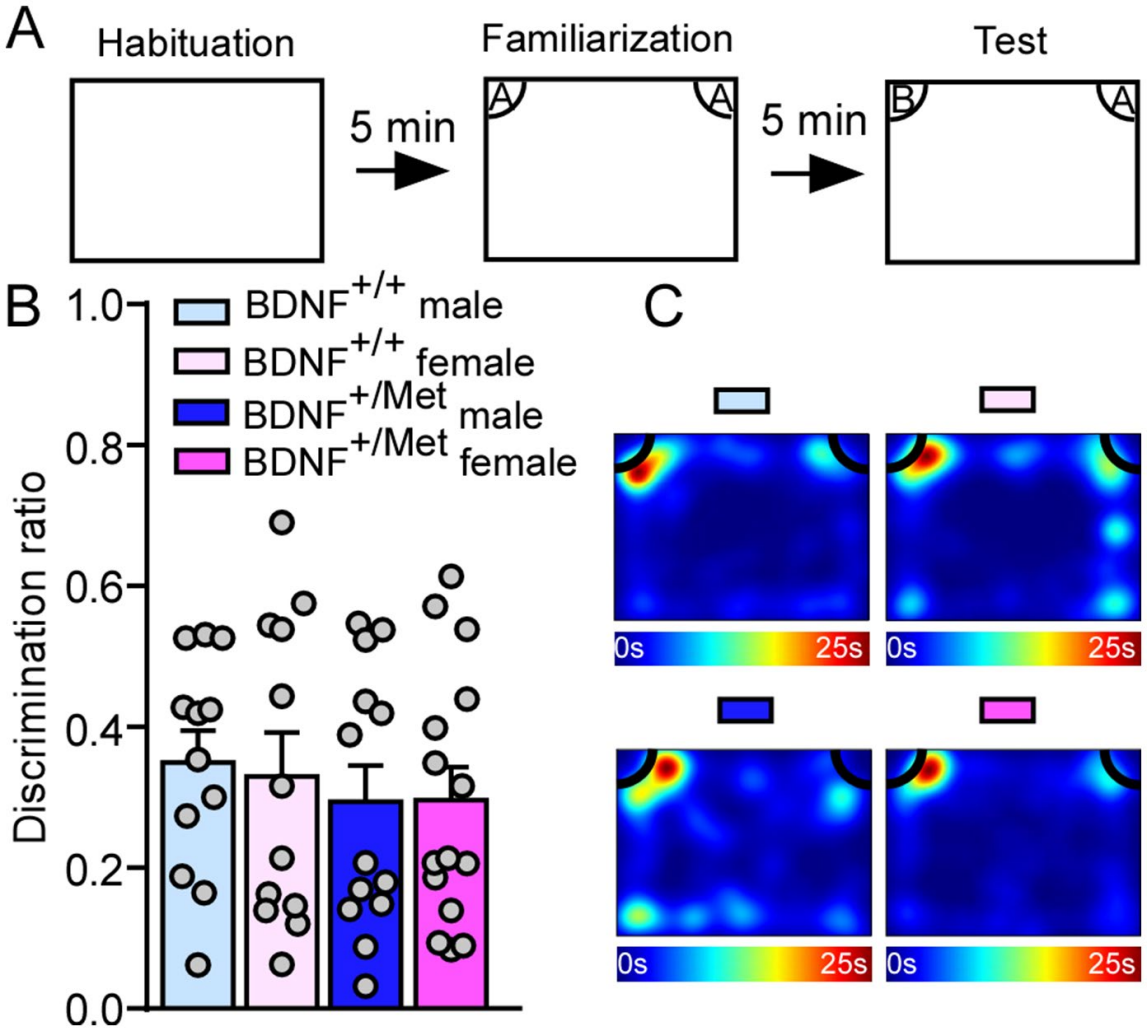
Diminished activity-dependent BDNF signaling does not affect object recognition memory in males and females. **(A)** Diagram showing the experimental procedure for novel object recognition test. **(B)** Bar graphs showing the discrimination index in NOR test of male and female BDNF^+/+^ and BDNF^+/Met^ mice. **(C)** Representative heatmaps showing the time spent exploring the familiar and novel object during the NOR test. Male BDNF^+/+^ mice: *n* = 12; Female BDNF^+/+^ mice: *n* = 12; Male BDNF^+/Met^ mice: *n* = 13; Female BDNF^+/Met^ mice: *n* = 15.

Human and animal studies have suggested a critical role for activity-dependent BDNF signaling in cognitive function (23, 24, 35). To assess the impact of diminished activity-dependent BDNF signaling on cognition, we did a NOR test to examine whether diminished activity-dependent BDNF signaling could impair object recognition memory. 5 min after initial familiarization with two identical objects in the habituated arena, the mice were allowed to explore the same arena in the presence of a familiar object and a novel object (Figure 3A). The results showed that male and female BDNF^+/Met^ ice had similar discrimination index with male and female BDNF^+/+^ mice (*F*_Genotype (1, 48)_ = 0.74, *p* = 0.39; *F*_Sex (1, 48)_ = 0.03, *p* = 0.87, Figures 3B,C), indicating diminished activity-dependent BDNF signaling has no effect on the object recognition memory.

### 3.4. Diminished activity-dependent BDNF signaling impairs spatial memory in female mice, but not in male mice

**Figure 4:**
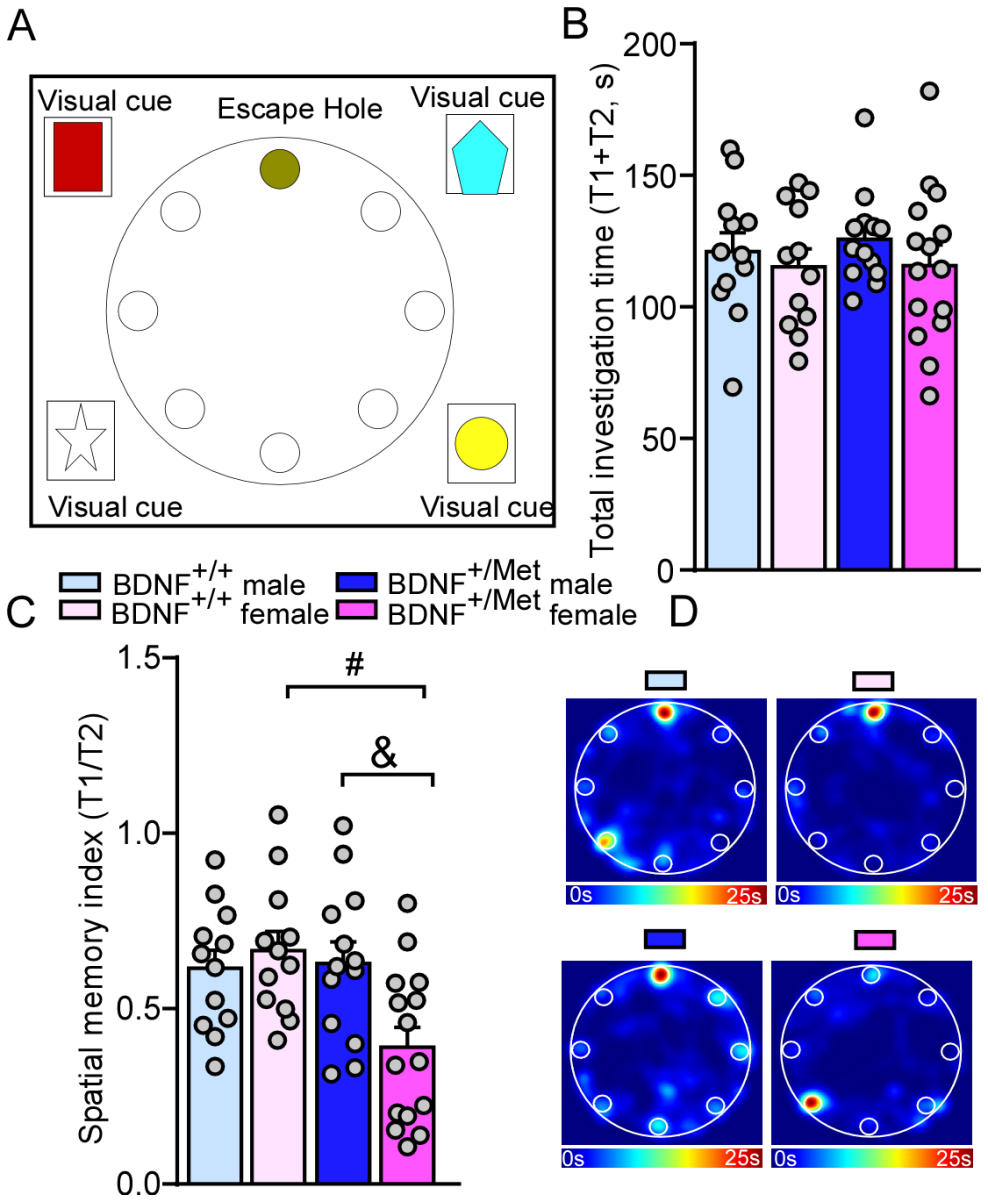
Diminished activity-dependent BDNF signaling causes spatial memory deficits in females, but not in males. **(A)** Diagram showing the scenario of Barnes maze test. **(B,C)** Bar graphs showing the total investigation time (T1 + T2) and spatial memory index (T1/T2) in Barnes maze test of male and female BDNF^+/+^ and BDNF^+/Met^ mice. **(D)** Representative heatmaps illustrating the time spent in different locations of the arena for Barnes maze tests during the memory phase (escape box removed). ^#^*p* < 0.05, BDNF^+/Met^ vs. BDNF^+/+^; ^&^*p* < 0.05, male versus female. Male BDNF^+/+^ mice: *n* = 12; Female BDNF^+/+^ mice: *n* = 12; Male BDNF^+/Met^ mice: *n* = 13; Female BDNF^+/Met^ mice: *n* = 15.

To determine whether diminished activity-dependent BDNF signaling affects spatial memory, we performed a Barnes maze test (26), which examines whether the mice remember the location of one correct hole (T1) (where an escape box was attached before) from seven other incorrect holes (T2) on a round platform (26, 36). Compared with male and female BDNF^+/+^ mice, male and female BDNF^+/Met^ mice spent similarly total investigation time (T1 + T2) on the target correct and incorrect holes (*F*_Genotype (1, 48)_ = 0.14, *p* = 0.71; *F*_Sex (1, 48)_ = 1.31, *p* = 0.26, Figures 4A,B). Surprisingly, female BDNF^+/Met^ mice displayed significantly lower spatial memory index (T1/T2), which was not shown in male BDNF^+/Met^ mice (*F*_Interaction (1, 48)_ = 6.42, *p* = 0.01; *F*_Genotype (1, 48)_ = 5.29, *p* = 0.03; *F*_Sex (1, 48)_ = 2.78, *p* = 0.1; *Post-hoc* Bonferroni’s multiple comparisons test: Adjusted *p* > 1.0 for male BDNF^+/+^ vs. female BDNF^+/+^ mice; Adjust *p* = 0.02 for male BDNF^+/Met^ vs. female BDNF^+/Met^ mice, Figures 4C,D). These results suggest diminished activity-dependent BDNF signaling has a sexually dimorphic effect on spatial memory.

## 4. Discussion

Valid animal models are essential for understanding the pathophysiology of ASD (37). In this study, we used mice with knock-in of a human BDNF Met allele to study the etiological role of diminished activity-dependent BDNF signaling in ASD. Given that males are more commonly diagnosed with ASD than females, we incorporated sex as a biological variable in this study to obtain further insight (38). We have provided the first pre-clinical evidence that diminished activity-dependent BDNF signaling induced autism-like behavioral deficits in both sexes, which supports that diminished activity-dependent neural signaling is a common molecular pathway in ASD. Furthermore, we have identified that diminished activity-dependent BDNF signaling differently affects the severity of autism-like behavioral deficits in males and females, revealing previously underappreciated sex-specific effect in ASD. These findings are consistent with the previous report that males are more vulnerable to having ASD (39, 40). Additionally, sex-specific spatial memory deficits were observed in only female BDNF^+/Met^.

The etiology of ASD remains elusive and is likely associated with both genetic and environmental factors. Epidemiologic studies have supported that genetic factor plays critical roles in ASD with higher concordance in monozygotic twins (41, 42). The high-risk genes for ASD have been identified mainly involved in regulation of gene expression and neuronal communication (1, 43). We and others have hypothesized that diminished activity-dependent neural signaling is a common molecular pathway converged by diverse genetic mutations (3, 4) and plays etiological roles in ASD. BDNF is synthesized and secreted in response to neuronal activity, and plays major roles in promoting neurogenesis, synaptic plasticity, and long-term potentiation in the brain (21, 22, 44). Recent studies have reported higher BDNF levels in the peripheral blood and brains of autistic patients than in healthy controls with unknown sexual effect (45–48), which indicate BDNF plays a critical role in the pathogenesis of ASD. Although significant progress has been made regarding the biological function of BDNF, the goal of this study is to determine the causal role of diminished activity-dependent BDNF signaling in ASD.

Anxiety is one of the comorbidities of ASD. Human BDNF Val66Met polymorphism has been linked to human psychiatric diseases including anxiety (23, 24, 49). Prior to investigating autism-like social behavioral deficits, we first examined whether BDNF^+/Met^ mice present anxiety-like behavior, which can help us accurately interpret the subsequent social behavioral results. Here, we have found that male and female BDNF^+/Met^ mice displayed decreased times spent in the center during the open field test, and decreased times and number of entries in the open arms during the EPM test, which demonstrated that diminished activity-dependent BDNF signaling induced anxiety-like behaviors. Our collaborators have shown that homozygous male and female BDNF^Met/Met^ mice displayed anxiety-like behavior in open field test (23, 31). Here, we first reported that heterozygous BDNF^+/Met^ mice displayed anxiety-like behaviors in both sexes, which demonstrates that the Met allele in the *BDNF* gene plays a key role in genetic predisposition to anxiety (23).

Social behavioral deficits are a hallmark of ASD. We found both male and female BDNF^+/Met^ mice displayed significantly decreased time spent with social stimulus and social preference index, which confirmed that diminished activity-dependent BDNF is a common pathway in males and females of ASD. The prefrontal cortex (PFC), a hub brain region, is critical for “high level” executive functions, including working memory, sustained attention, and social communication (50, 51), which is impaired in ASD patients and mouse models of autism (12, 18). The autism-like social behavioral deficits are likely linked to diminished activity-dependent BDNF signaling in PFC. PFC sends out glutamatergic transmission to downstream targets *via* top-down control, such as striatum, amygdala, nucleus accumbens (VTA) (18, 52). Also, PFC receives cholinergic and dopaminergic inputs from the basal forebrain and ventral tegmental area *via* bottom-up innervation (53, 54). The neural mechanisms underlying the sexual differences of diminished activity-dependent BDNF signaling on social deficits at circuit levels are complex, which need to be further investigated.

Striatum is a brain region important for stereotypic behaviors (55–57), and a region that expresses minimal BDNF and has been assumed to receive neurotrophic support *via* anterograde delivery of BDNF from the cortex (58). The elevated repetitive grooming behavior is primarily associated with diminished activity-dependent BDNF release from cortex to striatum, which indicates the causal link between PFC to striatum pathway and diminished activity-dependent BDNF signaling in increased self-grooming.

The autism-like social deficits and self-grooming were more severe in male BDNF^+/Met^ mice, which is consistent with the prevalence of ASD being higher in males than in females, at a about 4: 1 ratio (40, 59). Sexual hormones influence brain structure and function beyond classic reproductive function (60, 61), which makes males and females differently respond to similar challenges (62). The sexual differences in social behavioral deficits and self-grooming between male and female BDNF^+/Met^ mice indicate neuronal estrogen in the brain may contribute the milder autism-like behavioral deficits in female BDNF^+/Met^ mice, which needs to further be investigated. The frequency of SNP varies in humans, ranging from 20 to 30% in Caucasians and up to 70% in the Asian population (24, 63). The data from this preclinic mouse model are consistent with human studies showing significant association of BDNF Val66Met polymorphism in children with ASD in the Korean population (64). However, other studies failed to detect the significance of this SNP in autism in the Chinese population (65). A large sample size is required to determine whether the Met allele in the *BDNF* gene is a genetic marker in humans who are at increased risk for autism and males are more susceptible than females.

Activity-dependent BDNF expression is enriched in the hippocampus (66), which is a critical hub for social behaviors, memory, cognition and epilepsy, and is impaired in children with ASD (67, 68) and mouse models (32, 69). Humans carrying one Met allele have smaller hippocampal volumes and perform poorly on hippocampal-dependent memory tasks in males and females (24, 35). Histological data showed that diminished activity-dependent BDNF signaling caused decreased hippocampal volume and dendritic arbor complexity of granule cells in the dentate gyrus (DG) of male mice with unknown impact on female mice (23). Besides social deficits, cognitive impairments are another prominent phenotype in ASD patients and up to 70% of these patients have intellectual disability (70). While there was no difference in the novel object recognition test, when we compared male and female BDNF^+/Met^ mice to controls, which is consistent with other’s studies by using this mouse model (71). These results indicate that diminished activity-dependent BDNF signaling has mild effect on the brain regions related to object recognition memory formation such as perirhinal cortex (72). Interestingly, sexually dimorphic spatial memory impairments in the Barnes maze test were revealed in female BDNF^+/Met^ mice, but not in males, which is consistent with studies done by Marrocco et al. in a placement object task (73) and suggests females may be more vulnerable to spatial memory impairments. The sex-specific effect of diminished activity-dependent BDNF signaling on spatial memory was partially due to altered neuronal morphology the DG (23), impaired NMDA receptors-dependent synaptic plasticity (74) or sex-dimorphic regulation neuronal transcription in the CA3 (73).

In summary, diminished activity-dependent BDNF signaling resulted in autistic-like behaviors in both males and females with sexual difference in severity, and sex-specific spatial memory deficits in females, which demonstrated the construct and face validity of this knock-in of a human BDNF Met allele mouse model for ASD and built a strong basis for mechanistic studies. To support that diminished activity-dependent neural signaling plays a causal role in ASD, further studies to decipher the molecular, synaptic and circuitry mechanisms underlying autism-like behavioral deficits are required. These findings indicate a potential direction in therapeutic strategies to rescue autism-like behavioral deficits in patients with ASD. Drug discovery strategies to increase BDNF release from synapses or exogenous administration of drugs with similar function of BDNF may improve therapeutic responses for ASD patients.

## Data availability statement

The original contributions presented in the study are included in the article/Supplementary material, further inquiries can be directed to the corresponding author.

## Ethics statement

The animal study was reviewed and approved by the Institutional Animal Care and Use Committee of University of South Dakota. Written informed consent was obtained from the owners for the participation of their animals in this study.

## Author contributions

KM and LQ designed experiments, carried out the experiments, analyzed the data, interpreted the results, and wrote the manuscript. MW performed statistical power analysis. CT and SN edited the manuscript. LQ finalized the manuscript. All authors contributed to the article and approved the submitted version.

## Funding

This work was supported by startup funding of LQ from Sanford School of Medicine, University of South Dakota and Sanford School of Medicine Faculty Research Grant (2022). SN is supported by NIH MH106640. MW is supported by NIH NIGMS U54GM128729.

## Conflict of interest

The authors declare that the research was conducted in the absence of any commercial or financial relationships that could be construed as a potential conflict of interest.

## Publisher’s note

All claims expressed in this article are solely those of the authors and do not necessarily represent those of their affiliated organizations, or those of the publisher, the editors and the reviewers. Any product that may be evaluated in this article, or claim that may be made by its manufacturer, is not guaranteed or endorsed by the publisher.
