# Supplementary figures and images for: Diminished activity-dependent BDNF signaling differentially causes autism-like behavioral deficits in male and female mice
Source: Front Psychiatry. 2023 May 3;14:1182472. doi: 10.3389/fpsyt.2023.1182472 (PMC10189061; doi:10.3389/fpsyt.2023.1182472)

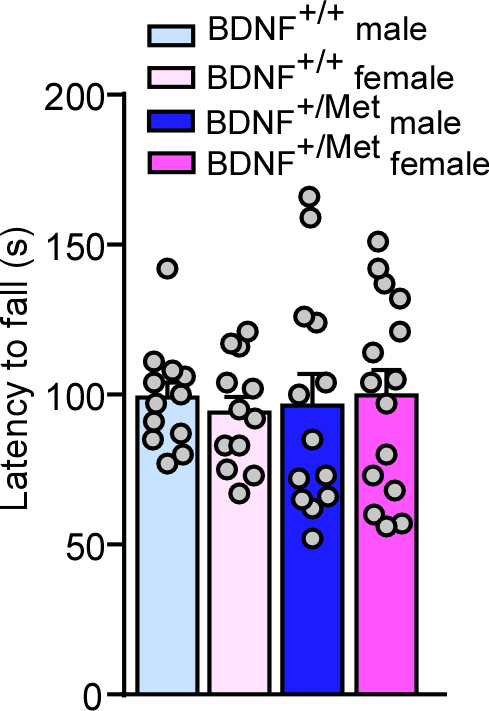

Supplement: Supplementary file 1 [file Image_1.TIF]

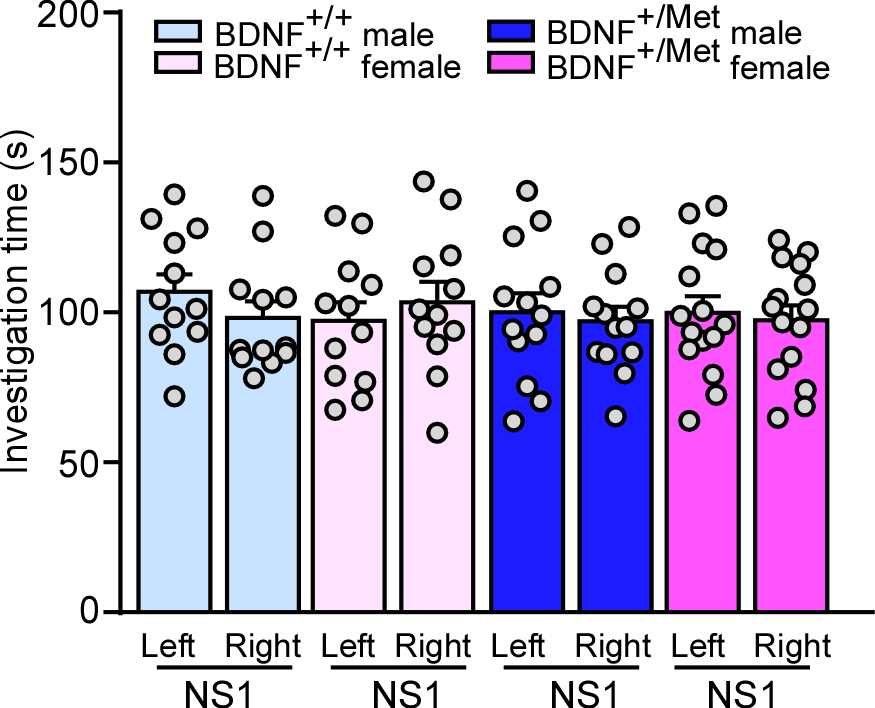

Supplement: Supplementary file 2 [file Image_2.TIF]
